# Targeting CHEK2-YBX1&YBX3 regulatory hub to potentiate immune checkpoint blockade response in gliomas

**DOI:** 10.1101/2025.03.09.642289

**Published:** 2025-03-13

**Authors:** Heba Ali, Ningjia Zhou, Li Chen, Levi van Hijfte, Vivekanudeep Karri, Yalu Zhou, Karl Habashy, Victor A. Arrieta, Kwang-Soo Kim, Joseph Duffy, Ragini Yeeravalli, Deanna M. Tiek, Xiao Song, Snehasis Mishra, Catalina Lee-chang, Atique U. Ahmed, Dieter Henrik Heiland, Adam M. Sonabend, Crismita Dmello

## Abstract

Although GBM’s immunosuppressive environment is well known, the tumor’s resistance to CD8+ T cell killing is not fully understood. Our previous study identified Checkpoint Kinase 2 (Chek2) as the key driver of CD8+ T cell resistance in mouse glioma through an in vivo CRISPR screen and demonstrated that Chk2 inhibition, combined with PD-1/PD-L1 blockade, significantly enhanced CD8+ T cell-mediated tumor killing and improved survival in preclinical model. Here, we aimed to elucidate the immunosuppressive function of Chek2. Immunoprecipitation (IP) followed by mass spectrometry (MS) and phosphoproteomics identified an association between Chek2 with the DNA/RNA-binding proteins YBX1 and YBX3 that are implicated in transcriptional repression of pro-inflammatory genes. Single-gene knock-out and overexpression studies of CHEK2, YBX1, and YBX3 in multiple glioma cell lines revealed that these proteins positively regulate each other’s expression. RNA sequencing coupled with chromatin immunoprecipitation-sequencing (ChIP-seq) analysis demonstrated common inflammatory genes repressed by CHK2-YBX1&YBX3 hub. Targeting one of the hub proteins, YBX1, with the YBX1 inhibitor SU056 led to degradation of CHK2-YBX1&YBX3 hub. Targeting of this hub by SU056 led to enhanced antigen presentation and antigen specific CD8+ T cell proliferation. Further, combination of SU056 with ICB significantly improved survival in multiple glioma models. Collectively, these findings reveal an immunosuppressive mechanism mediated by the CHK2-YBX1&YBX3 hub proteins. Therefore, CHK2-YBX1&YBX3 hub targeting in combination with immune checkpoint blockade therapies in gliomas is warranted.

## Full Text Availability

The license terms selected by the author(s) for this preprint version do not permit archiving in PMC. The full text is available from the preprint server.

